# The new normal for food insecurity? A repeated cross-sectional survey over 1 year during the COVID-19 pandemic in Australia

**DOI:** 10.1186/s12966-022-01347-4

**Published:** 2022-09-06

**Authors:** Katherine Kent, Sandra Murray, Beth Penrose, Stuart Auckland, Ella Horton, Elizabeth Lester, Denis Visentin

**Affiliations:** 1 School of Health Sciences, Western Sydney University, Campbelltown, Australia; 2 School of Health Sciences, University of Tasmania, Hobart, Tasmania Australia; 3 Tasmanian Institute of Agriculture, University of Tasmania, Hobart, Tasmania Australia; 4 Centre for Rural Health, University of Tasmania, Hobart, Tasmania Australia; 5 Institute for Social Change, University of Tasmania, Hobart, Tasmania Australia

**Keywords:** Food security, Food access, Food supply, COVID-19

## Abstract

**Background:**

Food insecurity during the COVID-19 pandemic has been impacted by necessary public health restrictions. Tasmania, an island state south of the Australian mainland, recorded no community transmission of COVID-19 between May 2020 to November 2021 due to strong border restrictions. This study aimed to determine the changes in prevalence and sociodemographic predictors of food insecurity throughout the COVID-19 pandemic in Tasmania, Australia.

**Methods:**

In May 2020 (survey 1: during lockdown), September 2020 (survey 2: eased restrictions) and May 2021 (survey 3: 1-year post-lockdown), cross-sectional, online surveys using convenience sampling methods determined food insecurity in Tasmanian adults using the USDA Household Food Security Survey Module: Six-Item Short Form, in addition to key sociodemographic questions. Crude and age-adjusted prevalence of food insecurity was calculated, and binary logistic regression determined at-risk groups and changes in prevalence over time.

**Results:**

The age-adjusted prevalence of food insecurity was 27.9% during lockdown (*n =* 1168), 19.5% when restrictions had eased (*n =* 1097) and 22.6% 1-year post-lockdown (*n =* 1100). Young adults, Aboriginal and/or Torres Strait Islander people, individuals with disabilities, families with dependents and temporary residents were at highest risk across all time points.

**Conclusions:**

The prevalence of food insecurity was higher than pre-pandemic levels across all three time points. Our results indicate the potential long-term impacts of the COVID-19 pandemic on food security in Australia, where despite easing social distancing restrictions and a lack of COVID-19 transmission, the prevalence of food insecurity reduced, but did not recover to pre-pandemic levels 1-year following a lockdown.

**Supplementary Information:**

The online version contains supplementary material available at 10.1186/s12966-022-01347-4.

## Background

As defined by the Food and Agriculture Organization of the United Nations, food security is a situation that exists “when all people, at all times, have physical, social and economic access to sufficient, safe and nutritious food that meets their dietary needs and food preferences for an active and healthy life” [1]. Food security comprises the concepts of appropriate availability, access (both physical and financial) and utilisation of food, in addition to the stability of these factors and the food supply. The COVID-19 pandemic and the necessary public health restrictions which were implemented by governments across the world to reduce the transmission of the virus have sparked widespread increases in global food insecurity [2], including in higher-income countries such as Australia.

The pathways through which the COVID-19 pandemic has impacted food security have been well described [3] and include the loss of jobs and income, mobility restrictions and social distancing disrupting the availability and access to food, social protections being threatened by political instability, and increasing conflict-driven food insecurity [3]. At the beginning of the pandemic in Australia, the availability of food was reduced as there was widespread panic buying of foods [4] and restrictions on the sale of staple food items that limited the availability of food [5]. Access to food was reduced through the closure of public transport, limits on the number of people from each household allowed to shop per day, and the closure of non-essential hospitality businesses and food markets. Financial access to food was reduced for many people who lost employment and income [6] and there was a corresponding increase in the number of people receiving government financial assistance (payments that fall well below the poverty line) [7]. Some people reported a reduction in the quality and safety of the food available [5] or that they lacked the skills and appropriate facilities to home-cook meals [8]. Finally, the stability of the national and global food supply was temporarily impacted [9]. As the period of instability in the food supply was relatively short in Australia, it is likely that food insecurity was primarily exacerbated by the indirect effects of public health restrictions such as mobility restrictions and reduced income [3].

A systematic review of food security research published in the first year of the pandemic demonstrated that the vast majority of studies reported that household food insecurity had increased and that food production and distribution was disrupted [10]. However, there was little published research from Australia or other Oceanian countries. We have previously reported the crude prevalence and sociodemographic predictors of food insecurity during the initial lockdown of the COVID-19 pandemic in Tasmania, Australia [11], where food insecurity was reported to be 26% using the U.S. Household Food Security Survey Module: Six-Item Short Form (HFSSM). The pre-COVID estimates of food insecurity in Tasmania was 6% in 2019 [12], indicating a substantial increase in food insecurity during this time. Multiple demographic groups were at higher risk, including young adults, people from Aboriginal and/or Torres Strait Islander backgrounds, people with a disability, people living in rural regions, people with less than a university-level education, temporary residents and households with dependent children [11]. We have also reported that food insecure households faced numerous challenges accessing food and employed many coping strategies, such as buying food on credit, during the beginning of the pandemic [5]. However, the persistence of food insecurity beyond the first few months of the pandemic has not yet been reported in Australia.

Fluctuations in the prevalence of food insecurity after the onset of the COVID-19 pandemic have been reported in the USA [13], showing a large increase in food insecurity followed by a partial recovery without returning to pre-COVID-19 levels. Large variations in food insecurity during periods of high COVID-19 case numbers or throughout lockdowns could also be evident in Australia, especially throughout the periods of strict social distancing restrictions and long lockdowns (see full overview of Australia’s public health response [14]). It is not known whether the observed increase in food insecurity at the beginning of the pandemic has returned to “normal”, nor whether the sociodemographic groups at-risk have changed. If increases in food insecurity during these periods are temporary, an understanding of how long it would take for food insecurity to return to pre-COVID levels is of substantial interest.

Given the vastly different trajectories of the COVID-19 pandemic around the world, evidence from a range of countries and contexts is required to inform responses to future pandemics that are appropriate for each setting. To fill a critical gap in research within an Australian context, this study aimed to compare the prevalence of food insecurity at three time points over 1 year during the COVID-19 pandemic in Tasmania, Australia. The following research questions guided our study:

- How did the prevalence and severity of food insecurity change between May 2020 to May 2021?
- Which population groups were at higher risk of food insecurity, and did these groups change between May 2020 and May 2021?

It was hypothesised that the prevalence of food insecurity would be highest at the beginning of the pandemic during a lockdown and significantly reduced after restrictions eased and 1-year post lockdown, but remain higher than pre-pandemic levels at subsequent timepoints (similarly to other high-income countries [14]). It was also hypothesised that discrete population groups would experience higher risk of food insecurity across all survey time points, which has been informed by our previous analysis of the sociodemographic predictors of food insecurity during the beginning of the COVID-19 pandemic [11], including:

1. younger adults compared with older adults due to surges in youth under- and un-employment during the pandemic;
2. people from Aboriginal and/or Torres Strait Islander backgrounds who experience greater disadvantage [15];
3. people with a disability compared with those without a disability, related to lower income and poorer access to food;
4. people living in rural areas compared with people living in urban areas due to supply chain challenges in rural regions;
5. people with less than a university-level education due to lower income;
6. temporary residents compared with citizens due to a lack of government financial protection available to this group during the pandemic; and
7. couple households with dependent children compared to couples without dependents due to higher expenses in these households.

## Methods

### Study setting

Tasmania is the island state of Australia sitting south of the Australian mainland, with a population of approximately 542,000 people [16]. Due to strict border and quarantine requirements, Tasmania, unlike other Australian states (such as New South Wales and Victoria), avoided large community outbreaks of COVID-19 and recorded no community transmission between May 2020 and December 2021. The COVID-19 timeline in Tasmania roughly follows the trajectory depicted in Table 1, which shows that after initial restrictions were enforced between March and June 2020, the Tasmanian community had been living in a COVID-free community without substantial social distancing restrictions affecting the availability of or access to food. In Australia, the Federal Government’s financial support mechanisms (JobSeeker – unemployment benefit and JobKeeper – wage subsidy further explained in Table 1), were available upon application to eligible Australians who had a documented reduction in income. These were the only national programs that addressed food insecurity for those who lost employment due to social distancing restrictions. Due to the lack of further coordinated response at a Federal level, the Tasmanian State Government provided additional funding to charitable agencies for scaling up emergency food relief. Due to challenges experienced with this model during the pandemic, state-level policies have prioritised transitioning toward more sustainable, place-based actions to support longer-term food insecurity such as school food programs [17].

**Table 1:** Timeline of COVID-19 restrictions in Tasmania, and relevant restrictions related to food access and supply (information sourced from: [18–21])

Date	Timeline
2nd March 2020	The Director of Public Health confirmed the first case of coronavirus in Tasmania.
17th March 2020	The Director of Public Health declared a Public Health Emergency for Tasmania.
19th March 2020	The Premier declared a State of Emergency under section 42 of the Emergency Management Act 2006. This included directions related to border restrictions, quarantine requirements and ‘stay at home’ requirements. Non-essential businesses, including food outlets were required to close.
2nd April 2020	The Premier announced additional restrictions, including the closure of farmers markets.
12th April – 1st May 2020	In response to an outbreak additional restrictions were imposed in specific areas of the North-West for 14 days with most retail businesses required to close.
27th April 2020	The Coronavirus Supplement was implemented for people receiving government income support payments (such as pensions and unemployment benefits – called JobSeeker), with a 61% increase in number of Tasmanian residents accessing JobSeeker payments between mid-2019 and mid-2020 as a result of the COVID-19 restrictions [21]. A wage subsidy program was also introduced for those retained by their employers but currently not working (JobKeeper). Figures of the number of JobKeeper recipients is not available.
18th May 2020	Stage One easing of restrictions, which included the opening of small food outlets for up to 10 patrons at a time.
5th June 2020	Stage Two easing of restrictions, which included intrastate travel, the opening of more businesses and food outlets able to seat up to 20 patrons at a time.
17th – 26th June 2020	Stage Three easing of restrictions. Markets, food outlets and food vans were able to open if they could maintain the “one person per 2 square metres” rule. Up to 250 people allowed to gather indoors and 500 outdoors. State borders remained closed to non-essential travellers without quarantine.
28th March 2021	The JobKeeper scheme ended.
31st March 2021	The Coronavirus Supplement was ceased and as a result unemployment (JobSeeker) and age-pension income support payments were reduced to slightly above pre-pandemic levels.

### Data collection

A series of surveys were conducted through *The Tasmania Project*, a longitudinal project established by the Institute for Social Change (University of Tasmania) to understand how Tasmanians are experiencing and adjusting to the social, political, and economic responses to COVID-19. In total, *The Tasmania Project* has conducted a series of 11 cross-sectional, online surveys since the beginning of the COVID-19 pandemic in Australia with a non-random sample of Tasmanian residents aged 18 years and over. Three surveys measured food security and have been included in this repeated cross-sectional measures analysis:

- Survey 1 - open from 25 May to 7 June 2020
- Survey 2 - open from 26 August to 6 September 2020
- Survey 3 - open from 29 April to 12 May 2021

With a recall period of the previous 30 days, Survey 1 captured experiences throughout and shortly after strict stay at home requirements (i.e., lockdown), Survey 2 captured experiences several months after most restrictions had been withdrawn (i.e., restrictions eased), and Survey 3 captured experiences approximately 1 year since the pandemic began and 4–6 weeks after a reduction in federal government financial support (see Table 1). For clarity, each survey period will be referred to in terms of the corresponding event at each time point: Survey 1: lockdown; Survey 2: restrictions eased; Survey 3: 1-year post-lockdown.

Participants were recruited to *The Tasmania Project* using a variety of convenience sampling methods including promoting the online survey through paid and unpaid social media posts, emailing research and community groups and advertising through print media and radio interviews. As participants were invited to sign up to a mailing list for future studies, many of the same participants are likely to have completed each of the three surveys, however data linkage across the studies was not possible due to limitations with the survey software and the scope of the ethics approval granted for the project. Due to the variety of recruitment methods, we are unable to determine the total number of people who received or saw the link for all three surveys, and as such we cannot calculate a response rate. However, we can report the number of participants on the mailing list who were sent a link to the survey via email and the number who clicked the link. We can also report the total number of participants directed to the survey via all recruitment methods who completed the screening questions, and those who completed the survey (qualified and answered at least one question). An overview of these figures for each survey is provided in Table 2. Participants used a generic link to enter each survey, which directed them to two qualifying questions to ensure they were aged 18 years and over and currently living in Tasmania, Australia. Participants were then provided with the participant information sheet and asked to provide consent by selecting “I have read and understood the Participant Information Sheet and I agree to take part in the project”. Eligible, consenting participants were able to complete the online, self-administered survey through SurveyMonkey.

**Table 2:** Description of the number of participants who entered the survey, consented, entered the survey and completed the USDA HFSSM 6 item tool [22]

		Survey 1: Lockdown (*n*)	Survey 2: Restrictions Eased (*n*)	Survey 3: 1-year post-lockdown (*n*)
Participants (mailing list only)	Sent email	1409	2232	2981
	Clicked survey link	683	653	734
Participants (the total number of participants directed to the survey via all recruitment methods)	Completed screening questions	1432	1301	1351
	Completed the survey (qualified and answered ≥1 question)	1432	1167	1176
	Completed the U.S. Household Food Security Survey Module: Six-Item Short Form [21]	1067	1133	1117

### Survey

At each of the three time points, food security status was determined using the HFSSM based on a reference period of the previous 30 days [22]. The HFSSM is a 6-item screening tool that has been validated against the longer 18-item USDA survey tool, showing this tool classified household food security status correctly in 97.7% of cases against the longer form [23]. The HFSSM comprises six questions that determine whether limited financial resources have led to inadequate food access, availability and utilization at a household level. Specifically, the HFSSM ask participants to provide an affirmative or negative response to the following questions: “The food that I bought just didn’t last and I didn’t have enough money to get more”, “I couldn’t afford to eat balanced meals”, “In the last 30 days did you ever cut the size of your meals or skip meals because there wasn’t enough money for food?” and if so, “How often did this happen?”, “In the last 30 days did you ever eat less than you felt you should because there wasn’t enough money for food?”, and “In the last 30 days were you ever hungry but didn’t eat because there wasn’t enough money for food?” [22]. In addition to the HFSSM, a range of sociodemographic questions were collected at all time points including gender (self-identified), age in years, Aboriginal and/or Torres Strait Islander status, whether they had a self-reported disability, their postcode, highest level of education, household composition and residency status.

### Statistical analysis

Data sets were exported from the online survey platform to IBM SPSS Statistics for Windows, version 26.0 (IBM Corp., Armonk, NY, USA) and prepared for statistical analysis which was performed in Stata 14.2 (Statacorp, 2015). All available survey data were used in the analyses. The significance level for all analyses was set at *p <* 0.05. Participants who did not complete the HFSSM were excluded from the analyses and participants who were missing values for a sociodemographic variable were excluded from analysis which included that variable.

Affirmative responses to the six questions on the HFSSM [24] were assigned a score of 1. Summed raw scores were used to categorize respondents as having high (0), marginal (1), low (2–4) or very low food security (5, 6). A binary variable was created where the high food security group were classified as “food secure”, and the marginal, low and very low food security groups were classified as “food insecure”, which is in line with recommendations from some research teams to classify marginal food security as being food insecure [25]. All sociodemographic variables were either categorical or ordinal and were cross-tabulated and summarized with frequencies and proportions. Crude prevalence rates of food insecurity were determined across all sociodemographic characteristics. As food security was commonly more prevalent amongst younger age groups and other sociodemographic factors related to age during the pandemic due to high unemployment and underemployment in this group during the pandemic, and as our sample had higher numbers of respondents in older age categories compared to the Tasmanian population, the crude prevalence of food insecurity could potentially underrepresent food insecurity. To account for this effect, the levels of food security status and the binary food security variable were adjusted for age using direct standardisation. Direct standardisation was performed using the 2020 ABS Tasmanian census data, with standardisation for each 5-year age group [26]. Univariate logistic regression was performed individually for each sociodemographic characteristic to generate unadjusted odds ratios for food insecurity using the crude prevalence statistics to determine the at-risk sociodemographic groups at each time point. Multivariable logistic regression was performed, including all measured variables to yield adjusted odds ratios (AOR) for food insecurity. Variables were included in an initial model and retained in the final model if any level of the variable had *p <* 0.1. Collinearity in the multivariate model building was assessed using the Variance Inflation Factor (VIF) with a scores > 5 indicating issues with collinearity. The change in prevalence over the three time points was determined using univariate and multivariate logistic regression adjusting for age, Aboriginal and/or Torres Strait Islander status, disability status, residency status, education, household composition, and rurality.

## Results

The sociodemographic characteristics (Table 3) demonstrate that the highest proportions of the study sample were aged 56 years and above, identified as female, were urban dwelling, had a university-level education, and were living in couple families with no dependent children. A minority of respondents were of Aboriginal and/or Torres Strait Islander descent and were living with a disability (Table 3).

**Table 3:** Sociodemographic characteristics of the study sample for Survey 1 (during lockdown), Survey 2 (after restrictions eased) and Survey 3 (1-year post-lockdown)

Demographics	Category	Survey 1: Lockdown n (%)	Survey 2: Restrictions Eased n (%)	Survey 3: 1-year post-lockdown n (%)
Age	18–25	28 (2.4%)	32 (2.8%)	32 (2.7%)
	26–35	117 (10.0%)	81 (7.2%)	66 (5.6%)
	36–45	201 (17.2%)	143 (12.6%)	134 (11.4%)
	46–55	235 (20.1%)	225 (19.9%)	208 (17.7%)
	56–65	266 (22.7%)	270 (23.8%)	281 (23.9%)
	65+	221 (18.9%)	267 (23.6%)	455 (38.7%)
Gender	Female	841 (71.9%)	746 (65.8%)	718 (61.0%)
	Male	249 (21.3%)	295 (26.0%)	345 (29.3%)
	Other	–	8 (0.7%)	13 (1.1%)
Aboriginal and/or Torres Strait Islander	Yes	25 (2.1%)	28 (2.5%)	32 (2.7%)
	No	1069 (91.4%)	1015 (89.6%)	1037 (88.2%)
Disability	Yes	238 (20.3%)	120 (10.6%)	335 (28.5%)
	No	857 (73.3%)	924 (81.6%)	736 (62.6%)
Rurality	Urban	792 (67.7%)	758 (66.9%)	759 (64.5%)
	Rural	306 (26.2%)	287 (25.3%)	312 (26.5%)
Education	University	737 (63.0%)	507 (44.8%)	736 (62.6%)
	Diploma/TAFE	211 (18.0%)	412 (36.4%)	213 (18.1%)
	High School	147 (12.6%)	129 (11.4%)	125 (10.6%)
Residency	Born in Australia	869 (74.3%)	806 (71.1%)	831 (70.7%)
	Born overseas, citizen	179 (15.3%)	200 (17.7%)	203 (17.3%)
	Permanent resident	32 (2.7%)	29 (2.6%)	32 (2.7%)
	Temporary resident	17 (1.5%)	12 (1.1%)	8 (0.7%)
Household status	Couple, no dependents	471 (40.3%)	497 (43.9%)	470 (40.0%)
	Couple, dependents	308 (26.3%)	221 (19.5%)	275 (23.4%)
	Single parent	65 (5.6%)	50 (4.4%)	25 (2.1%)
	Living alone	199 (17.0%)	215 (19.0%)	219 (18.6%)
	Other (group/share)	51 (4.4%)	74 (6.5%)	86 (7.3%)
All participants		*N =* 1067	*N =* 1133	*N =* 1176

**Fig. 1:**
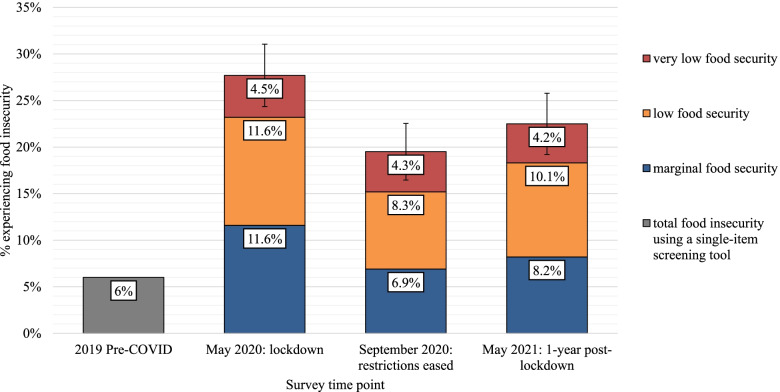
Age adjusted rates for food security categories: marginal, low, very low food security (error bars are 95% CI for total number of people who are “food insecure”) during lockdown, after restrictions eased and 1-year post lockdown. The 2019 pre-COVID prevalence statistic is estimated in a generalisable sample of the Tasmanian population using a single item food insecurity screening tool, which includes marginal, low and very low food security but is unable to define the severity of food insecurity experienced [12]. In May 2020, September 2020 and May 2021, food insecurity was determined using the USDA HFSSM 6-item tool [21, 22]

The crude prevalence for food insecurity according to all sociodemographic characteristics are provided in Supplementary Table 1. The crude and age-adjusted prevalence of food insecurity was 26 and 28% at Survey 1 during lockdown, 18 and 20% at Survey 2 when restrictions eased, and 18 and 23% at Survey 3 1-year post-lockdown (Table 3; Fig. 1). The age-adjusted prevalence of marginal food security was approximately halved between Survey 1 and 2 (12 to 7%) and then increased slightly at Survey 3 (8%). The age-adjusted prevalence of low food security was reduced by approximately one third between Survey 1 and 2 (12 to 8%) and then increased slightly at Survey 3 (10%), and very low food security stayed relatively unchanged between all three surveys (~ 4%) (Table 4; Fig. 1).

**Table 4:** Crude and age-standardised food security proportion estimates

	Food Security Status	Survey 1: lockdown *n =* 1067	Survey 2: restrictions eased *n =* 1133	Survey 3: 1-year post-lockdown *n =* 1117
Crude rates (% [95% CI])	Food Insecure (total)	26.1% [23.7, 28.7]	18.0% [15.8, 20.3]	17.8% [15.7, 20.1]
	Marginal food security	12.3% [10.6, 14.3]	6.2% [4.9, 7.8]	6.7% [5.3, 8.4]
	Low food security	10.1% [8.5, 12.0]	8.2% [6.7, 10.0]	8.2% [6.7, 10.0]
	Very low food security	3.7% [2.7, 4.9]	3.6% [2.6, 4.8]	2.9% [2.1, 4.1]
Age standardised rates (% [95% CI])	Food Insecure (total)	27.9% [24.4, 31.1]	19.5% [16.5, 22.6]	22.6% [18.3, 25.8]
	Marginal food security	11.6% [9.4, 13.8]	6.9% [4.9, 8.9]	8.2% [5.8, 10.5]
	Low food security	11.6% [9.0, 14.3]	8.3% [6.2, 10.5]	10.1% [7.6, 12.7]
	Very low food security	4.5% [2.8, 6.3]	4.3% [2.6, 4.9]	4.2% [2.4, 4.2]

The distribution of food insecurity between genders was similar at Survey 1 (26%), but higher in males at both Survey 2 and 3 (20% vs 16%; Table 5). The respondents in the youngest age category (18–29 years) had the highest prevalence of food insecurity in all three surveys, and the prevalence increased by nearly 12% (from 33% at Survey 1 and 2 to 44% at Survey 3) (Table 5). All other age groups experienced a reduction in food insecurity between Survey 1 and 2 of between 2 and 12%, and then between Survey 2 and 3 the prevalence for these age groups remained fairly similar (changing between 0.8 to 3.7%) (Table 5).

**Table 5:** Food insecurity proportion estimates –adjusted prevalence rates by age category and gender

	Survey 1: lockdown *n =* 1067		Survey 2: restrictions eased *n =* 1133			Survey 3: 1-year post-lockdown i1117			
	*n*	*p*	95% CI	*n*	*p*	95% CI	*n*	*p*	95% CI
**Gender**									
Female	841	25.5%	[22.6, 28.5]	746	16.4%	[13.9, 19.2]	718	16.6%	[14.0, 19.5]
Male	249	25.7%	[20.6, 31.5]	295	20.0%	[15.8, 25.0]	345	19.7%	[15.8, 24.3]
**Age**									
18–29	78	33.3%	[23.7, 44.6]	55	32.7%	[21.6, 46.2]	54	44.4%	[31.7, 57.9]
30–39	140	29.3%	[22.3, 37.4]	98	21.4%	[14.4, 30.7]	81	22.2%	[14.4, 32.6]
40–49	224	26.9%	[21.5, 33.1]	187	24.7%	[19.0, 31.5]	172	26.9%	[20.8, 34.1]
50–59	240	25.0%	[19.9, 30.9]	236	16.9%	[12.7, 22.3]	240	14.6%	[10.6, 19.7]
60–69	234	23.5%	[18.5, 29.4]	263	14.1%	[10.4, 18.8]	273	12.8%	[9.3, 17.3]
70+	152	20.4%	[14.7, 27.6]	179	8.4%	[5.1, 13.5]	239	12.1%	[8.6, 16.9]

*n* number of participants in each category, *p *adjusted prevalence rates, *95% CI *95% confidence interval

At each time point, univariate regression identified the sociodemographic factors associated with food security status (Table 6). At all three time points, gender was not significantly associated with food security status (Table 6). However, at every time point, younger adults, those with a disability, those of Aboriginal and/or Torres Strait Islander descent, those with lower levels of education, single parent households and temporary residents were at significantly increased risk of food insecurity (Table 6). Living in a rural area was significantly associated with food insecurity at Survey 1 and 2, but not 3. Couple families with dependent children and single person households were at significantly increased risk at Survey 3, but not 1 or 2. In the multivariate model, all variables remained significant predictors of food security status except for; single parent households at timepoint 1, level of education at timepoints 2 and 3, and both Aboriginal and Torres Strait Islander status and residency status at timepoint 3 (Supplementary Table 2). Collinearity was assessed in the model building with VIF < 5 for all variables in each of the models.

**Table 6:** Association between demographic factors and food insecurity—univariate logistic regression

		Survey 1: lockdown *n =* 1067					Survey 2: restrictions eased *n =* 1133			Survey 3: 1-year post-lockdown *n =* 1117			
Parameter	***Level***	OR	SE	p	95%CI	OR	SE	p	95%CI	OR	SE	p	95%CI
Gender	*Male*	1.01	0.167	0.943	[0,73, 1.40]	1.28	0.225	0.165	[0.90, 1.80]	1.22	0.206	0.231	[0.88, 1.70]
Age	*Increase in age per 10 years*	0.885	0.043	0.012	[0.81, 0.97]	0.752	0.422	< 0.001	[0.67, 0.84]	0.713	0.039	< 0.001	[0.64, 0.79]
Indigenous	*Yes*	3.68	1.586	0.001	[1.74, 8.64]	6.81	2.66	< 0.001	[3.16, 14.7]	2.58	0.983	0.013	[1.22, 5.44]
Disability	*Yes*	2.27	0.356	< 0.001	[1.67, 3.09]	3.86	0.80	< 0.001	[2.57, 5.80]	2.38	0.392	< 0.001	[1.72, 3.29]
Rurality	*Rural*	1.63	0.243	0.001	[1.22, 2.19]	1.82	0.311	< 0.001	[1.30, 2.54]	1.12	0.194	0.527	[0.79, 1.57]
Education	*University*	*Ref*				*Ref*				*Ref*			
	*Diploma/TAFE*	2.25	0.385	< 0.001	[1.61, 3.15]	1.36	0.240	0.078	[0.97, 1.93]	1.65	0.320	0.010	[1.13, 2.42]
	*High School*	2.56	0.494	< 0.001	[1.76, 3.74]	1.57	0.388	0.069	[0.97, 2.55]	1.96	0.449	0.003	[1.25, 3.07]
Household	*Couple, no dependents*	*Ref*				*Ref*				*Ref*			
	*Couple with dependents*	1.35	0.232	0.083	[0.96, 1.89]	1.41	0.315	0.121	[0.91, 2.19]	3.06	0.644	< 0.001	[2.02, 4.62]
	*Single parent household*	3.22	0.880	< 0.001	[1.89, 5.50]	5.32	1.67	< 0.001	[2.87, 9.85]	3.67	1.734	0.006	[1.46, 9.27]
	*One person household*	1.40	0.274	0.086	[0.95, 2.05]	1.55	0.341	0.048	[1.00, 2.38]	2.51	0.573	< 0.001	[1.61. 3.93]
Residency	*Born in Australia*	*Ref*				*Ref*				*Ref*			
	*Born overseas, citizen*	0.700	0.145	0.080	[0.47, 1.04]	0.846	0.185	0.443	[0.55, 1.30]	0.708	0.157	0.120	[0.46, 1.09]
	*Permanent resident*	1.37	0.537	0.421	[0.64, 2.96]	1.25	0.585	0.663	[0.50, 3.13]	0.632	0.343	0.397	[0.22, 1.83]
	*Temporary resident*	4,.11	2.05	0.005	[1.55, 10.9]	14.4	9.68	< 0.001	[3.84, 53.8]	4.42	3.154	0.037	[1.09, 17.9]

*OR* Odds Ratio, *SE* Standard Error, *p *p-value derived from univariate logistic regression, *95%CI *95% Confidence Interval

Univariate regression determined that the odds of food insecurity reduced by a factor of 30% between Survey 1 and 2 (*p <* 0.001), and by 19% from Survey 1 to 3 (*p =* 0.004). There was a non-significant increase in the prevalence of food insecurity of 16% between Survey 2 and 3, but this was not significant (*p =* 0.070). The multivariate analysis demonstrated the same trends. After adjusting for demographic characteristics of age, Aboriginal and/or Torres Strait Islander status, disability, residency status, education, household composition, and rurality, the odds of food insecurity were 34% lower at Survey 2 compared to Survey 1 (AOR:0.664; 95% CI [0.529, 0.834; SE:0.118, *p <* 0.001), but the odds of food insecurity were not significantly different between Surveys 2 and 3 (AOR:1.05; 95%CI: [0.804, 1.370]; SE-0.136; *p =* 0.720).

## Discussion

To the authors’ knowledge, this study of repeated cross-sectional surveys is the first in Australia to assess changes in the prevalence of food insecurity throughout the first year of the COVID-19 pandemic, and to determine the relationship with sociodemographic characteristics in a large sample of Australian adults. Our results indicate that the high levels of food insecurity documented at the beginning of the pandemic during a lockdown reduced but did not recover to pre-pandemic levels after 1 year, suggesting that a higher level of food insecurity may be the “new normal” in Tasmania, Australia. Our results are particularly concerning given that Tasmania is a state of Australia that was relatively unaffected by large-scale outbreaks of COVID-19 and avoided lengthy lockdowns relative to other regions of Australia and the world. As such, our results point to the potential for more substantial long-term impacts of the COVID-19 pandemic on food insecurity in Australia and globally, especially in regions with community transmission of COVID-19 and/or extended enforcement of public health restrictions.

There is a growing body of international research that shows that food insecurity has been exacerbated as a result of the COVID-19 pandemic and that the degree to which food security has been affected reflects both the extent of the spread of the virus and the severity of public health restrictions [10]. In our study, the age-adjusted prevalence statistics indicate that more than a quarter of Tasmanians experienced some degree of food insecurity during the initial lockdown at the beginning of the COVID-19 pandemic. During this time, strict social distancing restrictions [27] were coupled with challenges around food access and availability due to supply chain instability [28] and income reductions and job losses affecting financial access to food. Our previous research, which used the data from Survey 1 of this study, reported that these challenges disproportionately affected food insecure households [5], who were significantly more likely to be consuming less fresh food and have less food stored in their homes compared to food secure households. Despite the lack of community transmission of COVID-19 and easing of social distancing restrictions in Tasmania, the prevalence statistics only reduced slightly, to approximately 1 in 5 (or 20%) of households in September 2020 (Survey 2). This was several weeks after most restrictions were eased (Table 1), and the initial challenges related to food availability (e.g., panic buying) and physical access to food (e.g. shop closures) were largely resolved (Table 1). Additionally, while unemployment levels were still higher than prior to the pandemic [29], many individuals who had lost work, such as through the closure of non-essential retail and hospitality, had returned to work [30]. For unemployed individuals, the COVID-19 disaster relief payments were generally substantially lower than average wages [31], and thus likely to represent a reduction in income for some households, which may have restricted their financial access to food. Interestingly, the prevalence of food insecurity rose to 23% in May 2021, which may be related to the withdrawal of the federally funded Coronavirus Supplement which was paid to recipients of unemployment and other welfare payments, and to the ending of the JobKeeper scheme [30]. The withdrawal of these payments is likely to have further affected financial access to food for some households, as it has been documented that even prior to the pandemic people receiving government financial support payments struggled to afford food [32]. As the number of people receiving government financial assistance in June 2021 was 7.4% higher than March 2020, prior to the COVID-19 pandemic [7], it is likely that a large number of households, many of which were reliant on government assistance payments for the first time, had substantially reduced incomes 1 year after the initial lockdown.

Internationally, several studies have indicated similar trends in the prevalence of food insecurity during and after the initial lockdown. For example, in one study of families with dependent children conducted in the USA by Adams et al., (2021) [33], it was reported that the prevalence of food insecurity increased from 37% prior to the COVID-19 pandemic to 54% in May 2020, and then decreased to 45% by September 2020 without returning to pre-pandemic levels. A similar decline in food insecurity was reported in a study of adults in the USA using a validated two-item screening tool for household risk for food insecurity [34], with 54% of respondents classified at risk of food insecurity in April 2020, reducing to 41% by November 2020 [35]. The predictors of food insecurity were low income and living with dependent children [36]. The reduction in food insecurity several months after the onset of the pandemic globally could be related to the rebound of food supply chains which increased the availability of food, or to other social support responses implemented by governments and community groups such as emergency food relief. It is concerning that, even in Tasmania which did not have COVID-19 transmission in the community in the period of this study and was not been affected by extended lockdowns, the prevalence of food insecurity did not returned to pre-COVID-19 levels. Tasmania did, however, have higher levels of unemployment and underemployment due to its reliance on sectors such as tourism and hospitality, which were significantly impacted by strict border closures [37]. Ongoing monitoring of food insecurity should remain a priority to determine how long it will take, and what specific interventions are required, for prevalence statistics to return to “normal”. This should also be a priority for regions that have had long-term implementation of public health restrictions or disproportionately high levels of community transmission of COVID-19 in other states.

In our study, gender was not significantly associated with food security status, which is likely to be related to the fact that the surveys measured household food security and survey respondents were predominantly in couple households. However, our analysis shows there was a disproportionate burden in food insecurity experienced by young Tasmanians aged 18–29 years. The proportion of young people receiving unemployment payments doubled between March (5.6%) and May (11.5%) 2020, which was a higher increase than seen in other age groups and likely related to the loss of casual and temporary work during the pandemic. Our study also showed that food insecurity was substantially increased in May 2021 compared to earlier time points for this age group, which could be related to the withdrawal of the Coronavirus Supplement associated with the JobSeeker payment of which younger Australians were the primary recipients, but this requires further investigation [38]. There was a disproportionate economic disadvantage for young people during the pandemic because they were more likely to be unemployed or underemployed, and less likely to qualify for the JobKeeper scheme due to higher rates of casual work for this group [38, 39]. In our previous analysis, people receiving JobKeeper were not at higher risk of food insecurity compared to those with continuing employment, but people receiving the lower JobSeeker payment were at more than three times increased risk of food insecurity [11]. In a large study in the UK using a 2-item food insecurity screening tool, 3.2% of respondents reported being unable to eat healthy and nutritious food in the last week in April 2020, which increased to 16.3% in July 2020 [40]. They found that individuals who lost employment had an increased risk of food insecurity compared to individuals who were continuously employed or supported under the Coronavirus Job Retention Scheme [40], indicating that government financial support paid during the pandemic may have had a positive impact on food security [31]. However, the increased prevalence of food insecurity found in our study in May 2021, after the JobSeeker payment reduced in value and the JobKeeper payment was abolished may indicate that lowering government support payments could increase food insecurity for its recipients, which warrants further investigation [31]. In our study, temporary residents were at higher risk of food insecurity throughout the pandemic, which likely relates to the fact that they were excluded from receiving emergency COVID-19 government financial assistance [41].

In our study, lower levels of education were significantly associated with food insecurity at all time points. Education was also associated with food insecurity prior to the pandemic, however our results suggest it may have been exacerbated by this group having less access to the JobKeeper scheme, as they are more likely to be employed or be employed in casual jobs [42]. In addition, couple families with dependent children and single person households were at significantly increased risk at Survey 1 in May 2020. This aligns with international literature, with one study in the USA reporting that 14.7% of participants reported having low or very low food security, with higher prevalence (17.5%) among households with children. Further, unemployment, low education and low income were independently associated with higher odds of food insecurity among households with children [43], demonstrating the importance of financial access to food. Interestingly, in our study, rurality was significantly associated with food insecurity at Surveys 1 and 2, but not 3, which may relate to the fact that challenges with food security in rural areas are compounded by lower availability and access to healthy food when the food supply is disrupted. These results align with recent review of food insecurity in rural regions of high income countries during the COVID-19 pandemic [44] that demonstrated that food insecurity was often significantly higher in rural regions, which was often related to lower food availability and access to food during the pandemic. Given the stability of the food supply had recovered by May 2021, this could indicate that temporary food supply challenges disproportionately affected rural-dwelling Australians, extending previously published research [4].

A strength of our study is that age-standardised prevalence statistics were used to more accurately indicate the extent of food insecurity across the population of Tasmania. Further, our study includes a unique assessment of how food insecurity evolved over the COVID-19 pandemic that can be used to understand the longer-term impact of the pandemic and associated public health restrictions. The limitations of the study include the use of repeated cross-sectional surveys which precludes causality inferences, and the use of convenience sampling methods which may result in a response bias and limit the generalizability of our findings. As our surveys were online and in English, this may have resulted in a sample bias towards higher-literacy respondents with internet access. Despite standardising our estimates of food insecurity by age, our sample was overall more highly educated than the wider Tasmanian community, which may result in an underestimation of food insecurity given that education is protective against food insecurity [42]. The self-selecting nature of the sampling technique may have biased the results in ways that cannot be controlled for by the measured demographic variables. Our analyses are further limited by being unable to compare the prevalence of food insecurity by employment status or whether the participants received government financial support (such as JobSeeker and JobKeeper) throughout the pandemic, meaning we are unable to evaluate the importance of these factors for maintaining food security. Collecting this data should be a priority for future research, in addition to more comprehensive data on coping strategies and use of food relief programs. Lastly, we also lacked the ability to link survey respondents across time points, which limited our analysis as it is likely many of the same respondents answered all three surveys.

## Conclusion

In conclusion, our study determined the impact of the COVID-19 pandemic on the prevalence of food insecurity over 1 year in a sample of adults in Tasmania, Australia. Our results suggest that the prevalence of food insecurity was substantially increased at the beginning of the COVID-19 pandemic during a lockdown, and that the prevalence did not return to pre-COVID-19 levels 1 year after lockdown and when most public health restrictions had eased. Our results also suggest there may be long-term impacts of food insecurity for communities experiencing a high burden of COVID-19 infections or those which have experienced extended periods of strict public health restrictions such as lockdowns. Further, an increase in food insecurity after the withdrawal of government financial payments introduced because of COVID-19 suggests that young Australians may be particularly reliant on this support to afford food. The increased experience of food insecurity that we have described in this study is unlikely to return to pre-pandemic levels without intervention, such as targeted food solutions to support longer-term economic recovery for vulnerable groups such as young adults, temporary residents and families with dependent children. It is apparent that a transition from reliance on emergency food relief to community led, driven and owned food solutions is a priority in an attempt to avoid the statistics in our study from becoming the “new normal” for food insecurity in Tasmania, Australia. Consumers in this region who were affected by food supply chain disruptions during the pandemic have identified that multiple strategies to build resilience in the Tasmanian food supply should be a priority to maintain food security [45]. This includes balancing interstate and international food exports with local needs, strengthening local food systems by building collaboration and connections between food producers and consumers and advocating for well-funded initiatives that build a community’s capacity to respond to challenges with food insecurity, such as nutrition and food literacy programs that promote self-sufficiency for food production [45]. Our results provide further support for the urgent need for local food councils to be established by the Tasmanian government in partnership with the community, who would facilitate place-based community initiatives that strengthen local and sustainable food procurement to ultimately improve food security [46]. At a national level, the Australian government must expand initiatives that promote secure employment opportunities and support an increase in wages, especially for lower income workers. Raising the rate of government financial support payments has been suggested to be critical for reducing food insecurity in Australia [47]. Ongoing monitoring of the severity and demographic groups at risk of food insecurity must be a priority for both state and federal Governments to ensure a coordinated and targeted approach to addressing food insecurity across Australia.

## Supplementary Information

**Additional file 1: Supplementary Table 1.** Crude food insecurity prevalence statistics according to all demographic characteristics collected across the three surveys showing the n (%) of each row at the three timepoints (total column); and the n(%) of each row classified as food insecure at each timepoint (food insecure column). **Supplementary Table 2.** Association between demographic factors and food insecurity—multivariate logistic regression. (AOR = Adjusted Odds Ratio; SE = Standard Error; *p* = *p*-value derived from multivariate logistic regression; 95%CI = 95% Confidence Interval).

## Data Availability

Datasets are not publicly available due to ongoing analyses. However, datasets will be made available upon reasonable written request to the corresponding authors.
