# Supplementary material for: The new normal for food insecurity? A repeated cross-sectional survey over 1 year during the COVID-19 pandemic in Australia
Source: Int J Behav Nutr Phys Act. 2022 Sep 6;19:115. doi: 10.1186/s12966-022-01347-4 (PMC9449271; doi:10.1186/s12966-022-01347-4)
Supplement: Supplementary file 1 — Additional file 1: Supplementary Table 1. Crude food insecurity prevalence statistics according to all demographic characteristics collected across the three surveys showing the n (%) of each row at the three timepoints (total column); and the n(%) of each row classified as food insecure at each timepoint (food insecure column). Supplementary Table 2. Association between demographic factors and food insecurity—multivariate logistic regression. (AOR = Adjusted Odds Ratio; SE = Standard Error; p = p-value derived from multivariate logistic regression; 95%CI = 95% Confidence Interval). [file 12966_2022_1347_MOESM1_ESM.docx]

**Supplementary Tables**

Supplementary Table 1 – Crude food insecurity prevalence statistics according to all demographic characteristics collected across the three surveys showing the n (%) of each row at the three timepoints (total column); and the n(%) of each row classified as food insecure at each timepoint (food insecure column).

| **Demographics** | **Category** | **Survey 1: lockdown** | | **Survey 2: restrictions eased** | | **Survey 3: 1-year post-lockdown** | |
| --- | --- | --- | --- | --- | --- | --- | --- |
|  |  | **Total** | **Food Insecure** | **Total** | **Food Insecure** | **Total** | **Food Insecure** |
| Age | 18-25 | 28 (2.4%) | 12 (42.9%) | 32 (2.8%) | 12 (37.5%) | 32 (2.7%) | 17 (53.1%) |
|  | 26-35 | 117 (10.0%) | 37 (31.6%) | 81 (7.2%) | 17 (21.0%) | 66 (5.6%) | 15 (22.7%) |
|  | 36-45 | 201 (17.2%) | 55 (27.4%) | 143 (12.6%) | 38 (26.6%) | 134 (11.4%) | 38 (28.4%) |
|  | 46-55 | 235 (20.1%) | 60 (25.5%) | 225 (19.9%) | 44 (19.6%) | 208 (17.7%) | 35 (16.8%) |
|  | 56-65 | 266 (22.7%) | 61 (22.9%) | 270 (23.8%) | 41 (15.2%) | 281 (23.9%) | 49 (17.4%) |
|  | 65+ | 221 (18.9%) | 48 (21.7%) | 267 (23.6%) | 25 (9.4%) | 455 (38.7%) | 42 (9.2%) |
| Gender | Female | 841 (71.9%) | 214 (25.5%) | 746 (65.8%) | 122 (16.4%) | 718 (61.0%) | 119 (16.6%) |
|  | Male | 249 (21.3%) | 64 (25.7%) | 295 (26.0%) | 59 (20.0%) | 345 (29.3%) | 68 (19.7%) |
|  | Other | - | - | 8 (0.7%) | 3 (37.5%) | 13 (1.1%) | 4 (30.8%) |
| Aboriginal and/or Torres Strait Islander | Yes | 25 (2.1%) | 14 (56.0%) | 28 (2.5%) | 16 (57.1%) | 32 (2.7%) | 11 (34.4%) |
|  | No | 1069 (91.4%) | 264 (24.7%) | 1015 (89.6%) | 166 (16.4%) | 1037 (88.2%) | 175 (16.9%) |
| Disability | Yes | 238 (20.3%) | 92 (38.7%) | 120 (10.6%) | 48 (40.0%) | 335 (28.5%) | 89 (26.5%) |
|  | No | 857 (73.3%) | 186 (21.7%) | 924 (81.6%) | 136 (14.7%) | 736 (62.6%) | 97 (13.2%) |
| Rurality | Urban | 792 (67.7%) | 180 (22.7%) | 758 (66.9%) | 114 (15.0%) | 759 (64.5%) | 131 (17.3%) |
|  | Rural | 306 (26.2%) | 99 (32.4%) | 287 (25.3%) | 70 (24.4%) | 312 (26.5%) | 59 (18.9%) |
| Education | University | 737 (63.0%) | 146 (19.8%) | 507 (44.8%) | 76 (15.0%) | 736 (62.6%) | 110 (15.0%) |
|  | Diploma/TAFE | 211 (18.0%) | 75 (35.6%) | 412 (36.4%) | 80 (19.4%) | 213 (18.1%) | 48 (22.5%) |
|  | High School | 147 (12.6%) | 57 (38.8%) | 129 (11.4%) | 28 (21.7%) | 125 (10.6%) | 32 (25.6%) |
| Residency | Born in Australia | 869 (74.3%) | 224 (25.8%) | 806 (71.1%) | 139 (17.3%) | 831 (70.7%) | 153 (18.4%) |
|  | Born overseas, citizen | 179 (15.3%) | 35 (19.6%) | 200 (17.7%) | 30 (15.0%) | 203 (17.3%) | 28 (13.8%) |
|  | Permanent resident | 32 (2.7%) | 10 (31.3%) | 29 (2.6%) | 6 (20.7%) | 32 (2.7%) | 4 (12.5%) |
|  | Temporary resident | 17 (1.5%) | 10 (58.8%) | 12 (1.1%) | 9 (75.0%) | 8 (0.7%) | 4 (50.0%) |
| Household status | Couple, no dependents | 471 (40.3%) | 99 (21.0%) | 497 (43.9%) | 64 (12.9%) | 470 (40.0%) | 45 (9.6%) |
|  | Couple, dependents | 308 (26.3%) | 81 (26.3%) | 221 (19.5%) | 38 (17.2%) | 275 (23.4%) | 67 (24.4%) |
|  | Single parent | 65 (5.6%) | 30 (46.2%) | 50 (4.4%) | 22 (44.0%) | 25 (2.1%) | 7 (28.0%) |
|  | Living alone | 199 (17.0%) | 54 (27.1%) | 215 (19.0%) | 40 (18.6%) | 219 (18.6%) | 46 (21.0%) |
|  | Other (group/share) | 51 (4.4%) | 14 (27.5%) | 74 (6.5%) | 23 (31.1%) | 86 (7.3%) | 25 (29.1%) |
| All participants |  | *N* = 1067 | 305 (26.1%) | *N* = 1133 | 197 (17.4%) | *N* = 1176 | 196 (16.7%) |

Supplementary Table 2 Association between demographic factors and food insecurity—multivariate logistic regression.

|  | | |  | | | | **Survey 1: lockdown**  n = 1067 | | | | **Survey 2: restrictions eased**  n = 1133 | | | | **Survey 3:  1-year post-lockdown**  n = 1117 | | |
| --- | --- | --- | --- | --- | --- | --- | --- | --- | --- | --- | --- | --- | --- | --- | --- | --- | --- |
| **Parameter** | ***Level*** | **AOR** | | **SE** | **p** | **95%CI** | | **AOR** | **SE** | **p** | | **95%CI** | **AOR** | **SE** | | **p** | **95%CI** |
| Gender | *Male* | - | | - | - | - | | - | - | - | | - | - | - | | - |  |
| Age | *Increase in age per 10 years* | .886 | | .006 | .023 | [.974-.998] | | .792 | .007 | <.001 | | [.963-.990] | .708 | .007 | | <.001 | [.953-.978 ] |
| Indigenous | *Yes* | 2.776 | | .431 | .018 | [1.193-6.455] | | 4.509 | .430 | <.001 | | [1.942-10.468 ] | 1.224 | .426 | | .636 | [.531-2.821 ] |
| Disability | *Yes* | 2.130 | | .172 | <.001 | [1.521-2.983 ] | | 2.962 | .238 | <.001 | | [1.858-4.722 ] | 3.289 | .191 | | <.001 | [2.262-4.783] |
| Rurality | *Rural* | 1.658 | | .163 | .002 | [.1.206-2.281] | | 1.948 | .196 | <.001 | | [1.326-2.863 ] | - | - | | - | - |
| Education | *University* | *Ref* | |  |  |  | | *Ref* |  |  | |  | *Ref* |  | |  |  |
|  | *Diploma/TAFE* | 2.036 | | .183 | <.001 | [1.422-2.914] | | 1.413 | .200 | .085 | | [.954-2.093 ] | 1.633 | .214 | | .022 | [1.074-2.483] |
|  | *High School* | 2.222 | | .209 | <.001 | [1.475-3.349 ] | | 1.429 | .284 | .209 | | [.819-2.495 ] | 1.931 | .259 | | .011 | [1.163-3.205 ] |
| Household | *Couple, no dependents* | *Ref* | |  |  |  | | *Ref* |  |  | |  | *Ref* |  | |  |  |
|  | *Couple with dependents* | .936 | | .388 | .865 | [.437-2.003] | | 1.223 | .253 | .424 | | [.746-2.007] | 2.254 | .237 | | <.001 | [1.417-3.585 ] |
|  | *Single parent household* | 1.211 | | .379 | .613 | [.576-2.546 ] | | 3.947 | .355 | <.001 | | [1.968-7.915 ] | 3.419 | .503 | | .015 | [1.275-9.166] |
|  | *One person household* | 2.399 | | .448 | .051 | [.997-5.774] | | 1.516 | .256 | .104 | | [.918-2.503] | 2.047 | .246 | | .004 | [1.264-3.315] |
| Residency | *Born in Australia* | *Ref* | |  |  |  | | *Ref* |  |  | |  | *Ref* |  | |  |  |
|  | *Born overseas, citizen* | .858 | | .220 | .486 | [.558-1.319] | | 1.122 | .248 | .641 | | [.691-1.824 ] | .967 | .246 | | .891 | [.597-1.566 ] |
|  | *Permanent resident* | 1.346 | | .438 | .498 | [.570-3.178] | | 1.458 | .535 | .481 | | [.511-4.162 ] | .726 | .640 | | .616 | [.207-2.543] |
|  | *Temporary resident* | 5.168 | | .546 | .003 | [1.771-15.081] | | 13.397 | .718 | <.001 | | [3.277-54.767] | 4.534 | .848 | | .075 | [.860-23.904] |

(AOR = Adjusted Odds Ratio; SE = Standard Error; p = p-value derived from multivariate logistic regression; 95%CI = 95% Confidence Interval)
